# Peer Review of "Are We Sure We Fully Understand What an Infodemic Is? A Global Perspective on Infodemiological Problems"

**DOI:** 10.2196/39928

**Published:** 2022-07-21

**Authors:** Nabi Nazari

**Affiliations:** 1 Department of Psychology Lorestan University Khorramabad Iran

**Keywords:** communication, conspiracy, COVID-19, education, fake news, infodemic, infodemiology, mass media, public health, risk perception, science

*This is a peer-review report submitted for the paper “Are We Sure We Fully Understand What an Infodemic Is? A Global Perspective on Infodemiological Problems.”*

## Round 1 Review

Dear Authors,

This paper [1] presents a scientific and futuristic discourse on the context of infodemiology. However, I suggest arranging the content in order of importance. For example, I think the problem of predatory journals is overexplained. Moreover, the suggestion given regarding the mentioned problem is not practical, and the statements about the relationship between the editor and the referee do not seem fair. Additionally, the authors' statements about the duration of the submission review process are incomplete without an innovative result or proposal. In addition, the suggestions in the last part of the article need further explanation. Lastly, some of the items mentioned in the Abstract of the article have received little attention in the main text.
